# Role of adult neurogenesis in hippocampal-cortical memory consolidation

**DOI:** 10.1186/1756-6606-7-13

**Published:** 2014-02-19

**Authors:** Takashi Kitamura, Kaoru Inokuchi

**Affiliations:** 1 RIKEN-MIT Center for Neural Circuit Genetics at the Picower Institute for Learning and Memory, Department of Biology and Department of Brain and Cognitive Sciences, Massachusetts Institute of Technology, Cambridge, MA 02139, USA; 2 Department of Biochemistry, Faculty of Medicine, Graduate School of Medicine & Pharmaceutical Sciences, University of Toyama, 2630 Sugitani, Toyama 930-0194, Japan; 3 CREST, Japan Science and Technology Agency, Kawaguchi 332-0012, Japan

**Keywords:** Systems consolidation, Remote memory, Hippocampus, Synaptic plasticity, Neurogenesis

## Abstract

Acquired memory is initially dependent on the hippocampus (HPC) for permanent memory formation. This hippocampal dependency of memory recall progressively decays with time, a process that is associated with a gradual increase in dependency upon cortical structures. This process is commonly referred to as systems consolidation theory. In this paper, we first review how memory becomes hippocampal dependent to cortical dependent with an emphasis on the interactions that occur between the HPC and cortex during systems consolidation. We also review the mechanisms underlying the gradual decay of HPC dependency during systems consolidation from the perspective of memory erasures by adult hippocampal neurogenesis. Finally, we discuss the relationship between systems consolidation and memory precision.

## Introduction

The hippocampus (HPC) is crucial for the formation of episodic memories during the mnemonic process [1-5]. In memory-impaired humans and experimental animals (e.g., mice, rats, rabbits, cats, and monkeys), damage to the hippocampal structure predominantly affects recently acquired memories, whereas previously acquired old memories remain intact [1,6-9]. In rats, Kim and Fanselow [8] examined the temporal window during which the HPC is important for memory. This study was performed by applying electrolytic lesions to the HPC at various time points after contextual fear conditioning, an associative learning process that requires the HPC and the amygdale, between a chamber (context) and electrical footshocks that occur within that chamber [10,11]. Rats that were lesioned 1 day after training did not retain the contextual fear memory (1 day memory is considered recent memory), whereas animals that received the lesion 28 days later retained the memory (28 days memory is considered remote memory). This process is commonly referred to as systems consolidation theory [3,12-15]. Pharmacological inactivation studies using lidocaine and CNQX injections into the HPC or cortical regions support this concept [16-22]. Transgenic mice studies also support this concept. The αCaMKII heterozygous knockout mice showed the deficits in the cortical synaptic plasticity, but hippocampal synaptic plasticity was normal [23]. These animals showed an impairment of the remote memory retrieval, but the recent memory was intact. Similarly, forebrain-specific dominant-negative PAK transgenic mice showed the same phenotypes [24].

The systems consolidation process involves a time-limited interaction between the HPC and neocortical areas that eventually store long-term memory traces [22,25]. Studies monitoring the use of cerebral glucose [26], immediate early gene activation [16,17], and dendritic spine formation [22,27] have indicated that rapid on-line encoding of episodic memory in the HPC can be followed by temporally graded neural changes in the medial prefrontal (mPFC), orbitofrontal (Orb), anterior cingulate cortex (ACC), or retrosplenial (RSC) cortices. However, the mechanisms by which memory becomes progressively dependent on the cortical structure and independent of the HPC remain unknown. It is particularly important to determine 1) how the HPC and cortex communicate to consolidate the memory and 2) how the HPC dependency decays with time.

There is a central question regarding the complementary learning systems framework, as originally articulated by McClelland, McNaughton, and O'Reilly [28]. These authors asked “why is a hippocampal system necessary, if ultimately performance in all sorts of memory tasks depends on changes in connections within the neocortical system? Why does incorporation of new material into the neocortical system take such a long time?” In this review, we also discuss complementary learning systems from the perspective of memory precision and generalization.

## Mechanisms of cortical memory consolidation

There is an agreement among various memory systems consolidation models that interaction between the HPC and cortex (PFC, ACC, etc.) after the recent memory formation is crucial for the systems consolidation process [3,12-15,28]. However, little is known regarding which HPC circuit is important for this interaction between the HPC and neocortex. The entorhinal cortex (EC) and HPC neuronal networks contain two major excitatory circuits, the trisynaptic pathway (EC layer II/dentate gyrus/CA3/CA1/EC layer V) and the direct pathway (EC layer III/CA1/EC layer V), that converge onto a common HPC output structure, the CA1 region [29]. The CA1 output layer directly projects to several cortical areas (PFC, ACC, RSC, Orb) or through EC layer V to connect between the HPC and various cortical structures that eventually store long-term memory traces [30].

An initial experiment to investigate these connections was conducted by Remondes and Schuman [31]. These authors cut the ECIII-CA1 direct pathway by physical lesion and demonstrated that the lesion affected remote memories but not recent memories in spatial memory task and contextual fear memory task. However, more recently, Suh et al. [32] showed that triple transgenic mice, in which the output of the medial ECIII was specifically blocked by the expression of tetanus-toxin (TeTX) in medial ECIII, did not show any deficits in remote spatial memory. Thus, direct input from medial ECIII to CA1 may be not crucial for the systems consolidation process.

Nakashiba et al. [33] examined this discrepancy by taking advantage of the CA3-TeTX inducible transgenic mouse, in which a blockade of CA3 output was targeted only in the post-training period that followed contextual fear conditioning. They found that the blockade of CA3 output by TeTX during this period impairs the remote memory of contextual fear conditioning. Previous studies hypothesized that high-frequency field oscillations (“ripples”) in CA1 as well as the ripple-associated reactivation of experience-related firing patterns of CA1 pyramidal cells may be involved in systems consolidation [34-40]. Nakashiba et al. [33] showed that the intrinsic frequency of ripples and the reactivation process were both reduced significantly in the CA3-TeTX mice, supporting the hypothesis that these physiological processes are crucial for forming the cortical remote memory and thus are critical for the systems consolidation process.

Recently, Lesburgueres et al. [22] sought to identify the period during which the memory consolidation process is important for the interaction between the HPC and neocortex using chronic pharmacological inactivation. These authors examined the effects of repeated daily injection of CNQX into the HPC or the Orb after memory acquisition. The inactivation of HPC during days 1–15 (early period) after learning caused a deficit in the remote memory, whereas inactivation during days 16–30 (late period) after learning did not cause a deficit in the remote memory (30-day memory). This suggests that hippocampal activity during the early period is crucial for forming the remote memory. In contrast, the inactivation of the Orb either during early or late periods resulted in a deficit in the remote memory. Thus, hippocampal neuronal activity is transiently required (i.e., during the early period), although cortical activity is always required (i.e., during both the early and late periods). Interestingly, when the Orb was inactivated only during the training period, they found that an impairment in remote memory formation occurred without deficits in the 7-day memory [22]. This indicates that the neuronal activity of the Orb during the training period is required for the formation of remote memory but not recent memory, suggesting an early tagging by cortical networks for the consolidation of a memory into permanent cortical memory.

Based on these studies, it appears that information from networks of various neocortical regions is rapidly and temporarily linked through the HPC [22]. The HPC also activates the neocortex (ACC, PFC, Orb) during periods of inactivity and sleep via sharp-wave ripples (SPWs), during which connections between disparate cortical regions gradually develop [34-40]. Various studies have shown that the source of SPWs is synchronous with population bursts in the CA3 region of HPC [41-43] generated by its recurrent excitatory circuit [44,45]. Reactivation by the HPC serves to gradually strengthen the weak connections between neocortical sites. Eventually, the cortex can represent the memory of the original event in the absence of the HPC. Thus, the highly interconnected nature of the CA3 region may facilitate the integration of distributed memory fragments stored in the cortex to form the cortical permanent memory.

## Mechanisms for the decay of hippocampal dependency

During the systems consolidation process, a memory becomes progressively independent from the HPC. This decay of hippocampal dependency is thought to be an active process and plays a role in clearing old memories out of the HPC once the memory has been stored in the cortical networks, thereby allowing the HPC to continuously store new information [28]. Thus, neuronal systems could exist to erase the memory trace in the HPC.

The HPC is thought to be critical in the formation of the contextual component of episodic memories [30,46]. Contextual memory consists of the associations of objects/events and space (i.e., context), with medial EC and lateral EC inputs into the HPC providing spatial and object information, respectively [47]. Modeling and experimental studies have demonstrated that the dentate gyrus (DG) of the HPC has an essential role for discriminating between similar contexts [48-52]. Immediate early gene experiments showed that a sparse population of granule cells in the DG is activated in a given context, and different environments or different tasks activate different populations of granule cells [53-55]. Interestingly, the same sparse population of granule cells is activated repeatedly by the same environment. Recently, Liu et al. [56] used a strain of c-fos-promoter tTA transgenic mice [57,58] with the delivery of channelrhodopsin-2 (ChR2) into the DG by adeno-associated virus to directly demonstrate that a set of contextual memory-related DG cells that are optogenetically activated during the learning period is sufficient to recall the contextual memory without a conditioned cue. These lines of evidence indicate that the DG encodes the contextual memory engrams that represent discrete environments.

In the DG, new neurons are continuously generated in the subgranular zone throughout adulthood in many mammals, including mice, rats, monkeys, and humans, even during old age [59-62]. The newly generated neurons form synapses and are functionally integrated into existing hippocampal neuronal circuits [63-67]. The level of adult hippocampal neurogenesis is positively and negatively modulated by environmental conditions, neuronal activity, aging, and stress [68-77]. Most research has focused on the functional roles of neurogenesis in memory acquisition and in the early period of memory formation [71,78-83]. Theoretical studies suggest that the continuous insertion of newborn neurons into existing adult circuits could potentially disturb the structure of previously stored context information in the dentate gyrus [69,84]. Feng et al. [85] generated forebrain-specific presenilin-1 knockout mice that exhibited a deficiency in enrichment-induced neurogenesis in the DG. Although exposure to an enriched environment for 2 weeks decreased the freezing responses in contextual fear conditioning in wild type mice, the knockout mice did not show decreased freezing, suggesting a role for adult neurogenesis in hippocampal memory clearance.

**Figure 1:**
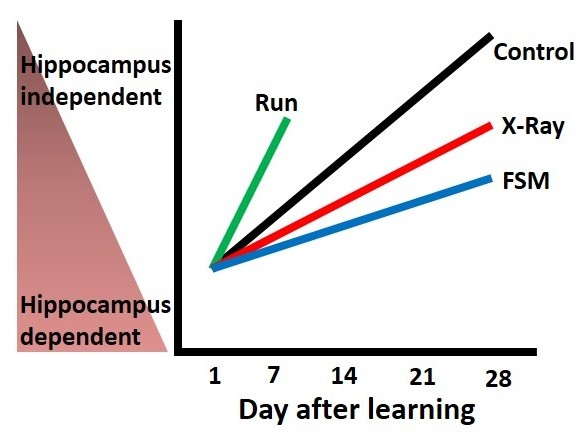
**Adult neurogenesis modulates the hippocampus dependent periods of memory.** The vertical axis shows the HPC dependence of contextual fear memory. The horizontal axis shows the days after learning. The HPC dependence of contextual fear memory gradually decays with time in control mice. In contrast, X-ray irradiation or genetic overexpression of follistatin (FSM), both of which severely impair hippocampal neurogenesis, prolong the HPC-dependent periods of contextual fear memory, when compared to control mice. Conversely, the running wheel exercises, which promote hippocampal neurogenesis, speed up the decay rate of the HPC dependency of contextual fear memory, when compared to control mice.

Kitamura et al. [19] examined whether the level of hippocampal neurogenesis affects the HPC-dependent periods of recall of contextual fear memory via a transient pharmacological inactivation of hippocampal neuronal activity (Figure 1). Decreased adult neurogenesis, either by X-ray irradiation or suppression of activin signaling [86], results in a prolonged HPC-dependent period of contextual fear memory (Figure 1). Thus, continuous integration of newborn neurons disturbs contextual memory in the dentate gyrus.

Decreased neurogenesis by irradiation also enhances the long-term retention of long-term potentiation (LTP; [87]) of the rat dentate gyrus, indicating that the synaptic integration of new neurons into existing neuronal circuits actively interferes with LTP persistence, which in turn leads to LTP decay [19]. LTP is a neural activity-dependent, long-lasting enhancement in synaptic efficacy, a typical form of synaptic plasticity [88]. LTP decay in the DG is an active, gradual process that is mediated by the N-Methyl-d-aspartic acid (NMDA) receptor; hence, daily administration of an NMDA receptor antagonist inhibits LTP decay following LTP induction [89]. There is a strong correlation between LTP and learning and memory. Physiological, pharmacological, and genetic interventions that alter or occlude LTP are accompanied by impairments in learning and memory maintenance [4,90]. Furthermore, HPC-dependent learning induces LTP in the HPC [91]. Thus, learning-induced LTP in the dentate gyrus may be gradually reversed by adult neurogenesis, similar to tetanus-induced LTP. This notion implies that the hippocampal memory trace is potentially lost due to interference brought on by neurogenesis. This body of evidence leads us to predict that the gradual decay of the HPC dependency of memory reflects the gradual erasure of the hippocampal memory trace mediated by hippocampal neurogenesis.

Electron microscopic examinations have also suggested that synaptic competition between old and new neurons occurs when newborn neurons form synaptic connections with pre-existing boutons in the DG [65,66]. Specifically, newly generated neurons transiently (at approximately 2–6 weeks of cell age) have enhanced synaptic plasticity, suggesting that at approximately 2–6 weeks of age, newly generated neurons may also have a strong ability to deprive pre-existing synapses of the presynaptic boutons [92,93]. Ohkawa et al. [67] also suggested that the integration of newborn neurons contributes to activity dependent synaptic rewiring in the dentate gyrus. Thus, the integration of newly born neurons disturbs the pre-existing circuits in the dentate gyrus, which may induce memory clearance from the HPC.

Consistent with our idea, Frankland et al. [94] also develop the hypothesis that ongoing hippocampal neurogenesis represents a decay process that continually clears memories from the hippocampus. They proposed, as an anterograde impact, ongoing neurogenesis may work for facilitated acquisition of memory. As retrograde impact, ongoing neurogenesis may work for more forgetting [94].

Kitamura et al. [19] showed that enhanced neurogenesis, resulting from running wheel exercise, hastens the decay of the HPC dependence of memory without any loss of memory (Figure 1). Consistent with this observation, exposure of animals to environmental enrichment accelerates the decay of LTP in dentate gyrus [95] while at the same time enhancing hippocampal neurogenesis [96]. These results imply that the recall of a memory depends on extra-HPC components acting in concert with the decay of HPC-dependency; otherwise, the memory would simply be lost. This interpretation leads us to predict that there may be a coupling mechanism between the decay of HPC-dependence and the increase in neocortex-dependence over time.

**Figure 2:**
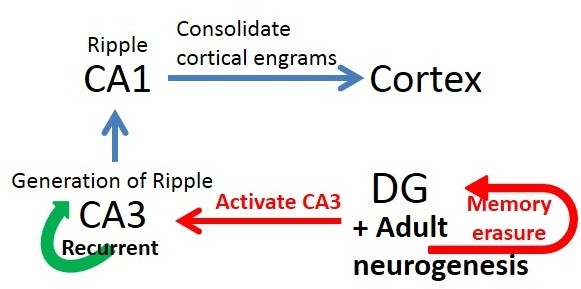
**How does adult neurogenesis accelerate the systems consolidation?** Adult hippocampal neurogenesis in the DG may help to generate the SPWs in CA3. The SPWs could provide the activation required to drive inter-cortical plasticity through the reactivations of CA1 pyramidal cells, and therefore promote cortical consolidation during subsequent periods of inactivity and sleep. Simultaneously adult neurogenesis erases the pre-exciting memory in the DG.

How does adult neurogenesis contribute to the acceleration of shift of memory dependency from HPC to cortical structures? One possibility is that adult hippocampal neurogenesis in the DG helps to generate the SPWs in CA3 [34-40]. The SPWs could provide the activation required to drive inter-cortical plasticity and therefore promote cortical consolidation during subsequent periods of inactivity and sleep. In addition, it has been found that the DG can control gene expression in various cortical regions during periods of sleep [97] (Figure 2). Thus, adult neurogenesis in the DG may have two distinct roles in systems consolidation process: 1) erasing the old memories in the HPC to maintain the storage capacity for new memories, and 2) regulating systems consolidation. Adult neurogenesis in the DG decreases with age, which is associated with an age-related decline in spatial memory [98-100]. Given the finite storage capacity of the DG, reduced neurogenesis in aged animals may limit the capacity of the HPC to acquire and store new information by reducing the clearance of old memories that have already been stored in cortical networks. On the other hand, Josselyn and Frankland [101] proposed that extensive early postnatal neurogenesis in the dentate gyrus could explain the biological mechanisms of infantile amnesia.

## Memory age vs. Memory precision or generalization

It has been suggested that the quality of original memories transforms from a precise (i.e., detailed) form to a less precise (i.e., more schematic or generic) form with a time course similar to that of the systems consolidation process [102-104]. Sparse inter-cortical connections may degrade some of the less well-represented content of the original memory, making remote memories more general and semantic-like in nature. Knowledge of this process is important for understanding the physiological significance of the hippocampal-cortical complementary memory systems. Using a contextual fear conditioning paradigm, studies [20,105] have demonstrated that the HPC is always necessary for the precision of place memories, supporting the memory transformation concept [103] in which the quality of place memory correlates with the brain region on which that memory depends. In contrast, another study [106] demonstrated that the HPC is not required for memory precision after the passage of time, supporting the memory reorganization concept [107] in which the quality of place memory does not correlate with the brain region. Importantly, this discrepancy can be attributed to differences in experimental protocols used for association with fear [108,109]. Because association with fear modifies (i.e., strengthen or generalize) the precision, we cannot rule out the possibility that fear association may mask the actual precision of place memory.

To directly evaluate context memory without using fear association protocols, Kitamura et al. [110] examined a one-trial and non-associative place recognition test in mice, and subjected the mice to the remote place memory test (28-days) with or without pharmacological inactivation of the HPC. In the remote memory test, even in the inactivation conditions, mice showed adaptation to a remotely experienced place but not to a novel place (i.e., mice discriminated a known place from a novel place), indicating that the inactivation of the HPC does not inhibit the precision of remote context memory. Thus, the contextual memory is precisely maintained for one month, although the recall of context memory shifts from being HPC-dependent to being HPC-independent with time. This indicates that an extra-HPC structure carries the intact information for context memory even after being independent from the HPC without any loss of context memory precision. Thus, one of the benefits of complementary learning systems could be the maintenance of the precision of memory quality. This is consistent with the idea that a rapid integration of arbitrary new information into neocortical structures would be avoided to prevent catastrophic interference with structured knowledge representations stored in synaptic connections among neocortical neurons [25,111].

### Future directions

Considering the mechanisms of systems consolidation of contextual fear memory from the perspective of the levels of memory engram [15,56-58,112,113], several fundamental questions remain. (1) Is the context memory engram in the DG actually erased (or just unused) after the completion of systems consolidation by newly generated neurons? This is very important question, because there are also reports that the HPC is still needed to retrieve the memory even after systems consolidations (for example, the context-dependent food preference memory is always required the hippocampus) [102-105,114]. Goshen et al. [21] also suggest the optogenetic inhibitions of CA1 pyramidal cells inhibited the retrieval of remote contextual fear memory (This discrepancy could be due to the compensation effect or cell-type specific manipulations). (2) How would context memory be represented in a cortical structure (in PFC or ACC) after systems consolidation to precisely maintain the information? To directly address these two questions, promising methodologies include a recently published transgenic approach [115] in which the expression of CreER, a tamoxifen-dependent recombinase, is under the control of an activity-regulated promoter, such as c-fos or activity-regulated cytoskeleton-associated protein (Arc) promoters. This approach may allow for permanent labeling of the memory-related cells in the HPC, cortex, and amygdala and allow for the manipulation of the activity of these cells to evaluate whether these cells remain indispensable for the retrieval of remote memory.

Although pharmacological and lesion approaches continue to be very useful for testing memory dependency on the HPC and cortical regions, an optogenetic approach using transgenic mice combined with viral technologies, in which the manipulation of cellular activity is more restricted to a specific population of memory-related cells, allows improved anatomy- and memory engram-based analyses and is better suited to understanding the mechanisms of the systems consolidation process.

## Competing interest

The authors declare that they have no competing interests.

## Authors’ contributions

TK and KI prepared the manuscript. Both authors read and approved the final manuscript.
